# The pursuit of happiness: Unraveling its key determinants among nursing students in Pakistan

**DOI:** 10.12669/pjms.42.6.13622

**Published:** 2026-06

**Authors:** Saba Khurshid, Ayesha Abubakar Mitha, Ambreen Fatima, Atia Suhail

**Affiliations:** 1 Saba Khurshid, Department of Research and Development, National University of Medical Sciences, Rawalpindi, Pakistan; 2 Ayesha Abubakar Mitha, Department of Healthcare Administration, Armed Forces Postgraduate Medical Institute, Rawalpindi, Pakistan; 3 Ambreen Fatima, Armed Forces Institute of Mental Health, Rawalpindi, Pakistan; 4 Atia Suhail, Armed Forces Institute of Mental Health, Rawalpindi, Pakistan

**Keywords:** Academic Performance, Happiness, Mental well-being, Nursing students, Social support

## Abstract

**Objective:**

This study aimed to investigate the determinants of happiness among nursing students in Pakistan by examining the associations between happiness levels and demographic, psychosocial, institutional, and lifestyle factors.

**Methodology:**

A cross-sectional study was conducted with 302 nursing students between April 18^th^ to June 30, 2025 in the nursing colleges located in Rawalpindi and Islamabad. Data were collected using a structured questionnaire based on two sections: demographic profile and Oxford Happiness Questionnaire Short Form (OHQ-SF) to assess happiness level. Descriptive statistics, chi-square tests, and ordinal logistic regression were used for data analysis.

**Results:**

A total of 302 nursing students participated in the study, with the majority being female (87.4%) and single (97.7%). Happiness among students declined with academic progression, particularly in the second year (p = .003). Higher happiness was significantly associated with strong faculty support, good sleep, satisfaction with nursing education, a positive work environment, access to mental health services, and an active lifestyle (all p < .05). Ordinal regression confirmed that personal choice of profession, moderate physical activity, and part-time employment were key predictors.

**Conclusion:**

The happiness among nursing students is related to individual characteristics, institutional environment, personal choice and healthy lifestyle. Therefore, there is need for nursing programs to support student autonomy, healthy lifestyles and institutional satisfaction to strengthen emotional resilience and well-being among nursing students in Pakistan.

## INTRODUCTION

Happiness, often regarded as a subjective state of well-being and life satisfaction, plays a vital role in the mental and emotional health of individuals. In recent years, it has attracted considerable attention in psychological, sociological, and public health research, serving as both a personal and societal aim.^1^ For healthcare professionals and students, particularly those in nursing, happiness transcends a personal ideal; it is intricately linked to academic performance, professional effectiveness, and quality of care provided.^2^ Nursing students encounter a unique set of challenges that can significantly impact their happiness and well-being.^3^

These challenges encompass the demanding nature of clinical placements, the emotional toll of patient care, financial difficulties, and pressure to meet high academic standards.^4^ The cumulative stress from these experiences can lead to anxiety, burnout, and diminished life satisfaction, ultimately hindering the personal and professional growth of the nurses.^5^,^6^ In such a high-pressure environment, happiness serves as a protective factor and psychological resource that fosters resilience, motivation, and academic success.^7^,^8^

Extensive literature underscores happiness multifaceted nature. Studies from diverse cultural contexts suggest that factors such as perceived social support, personal resilience, self-efficacy, financial stability, and institutional environments are key determinants of happiness.^9^,^10^ For instance, Özyeşil et al., Tembelo et al.^11^ argued that positive social relationships buffer against stress, whereas financial well-being enhances autonomy and reduces anxiety. Similarly, personal traits such as optimism, emotional intelligence, and coping mechanisms have consistently been associated with higher happiness scores among university students.^12^ However, much of the existing evidence originates from Western and East Asian educational contexts, with limited empirical focus on South Asian countries, such as Pakistan.

In Pakistan, nursing students often face additional stressors rooted in sociocultural norms, gender roles, institutional hierarchies, and resource limitations within the healthcare education system.^13^,^14^ These culturally embedded factors may influence the relationship between known predictors of happiness and actual outcomes, necessitating context-specific research to explore these relationships. Moreover, societal attitudes toward nursing in Pakistan, often shaped by gendered expectations and limited professional prestige, may affect students’ self-esteem, career satisfaction, and, ultimately, their emotional well-being.^5^ The lack of mental health support systems within academic institutions further exacerbates these challenges, making it crucial to examine how nursing students navigate their academic and personal lives to achieve happiness.^15^

Therefore, this study aimed to investigate the key determinants of happiness among nursing students in Pakistan by integrating personal and environmental variables into the analysis. By identifying these factors, this study seeks to inform institutional strategies that foster emotional resilience, promote mental well-being, and enhance the overall educational experience.

## METHODOLOGY

A cross-sectional study was conducted among undergraduate nursing students enrolled at the College of Nursing main campuses located in Rawalpindi and Islamabad, Pakistan, from April 18^th^ to June 30^th^ 2025. The students were eligible if they were in their second, third, or fourth year, or their internship period, have received clinical training, and consented to participate voluntarily. Whereas, students who were on academic leaves and who joined during academic session were excluded from study. A total 400 students were approached and 302 students completed the questionnaire, yielding a response rate of 75% by using a convenience sampling technique. Data were collected using a structured questionnaire comprising two main components. The first component was a sociodemographic profile sheet designed to gather essential background information from the participants, such as age, gender, year of study, type of institution (public or private), marital status, family income, and residential status (hostel or home). It also included lifestyle-related items, such as average sleep duration, self-reported sleep quality, and perceived social support from family, peers, and academic mentors.

The second component of the instrument was the Oxford Happiness Questionnaire Short Form (OHQ-SF), developed by Hills et al. and Argyle et al.^16^ The level of happiness among participants was assessed using an eight item subjective happiness scale. Each item was scored on a 6-point Likert scale, with options ranging from one (strongly disagree) to six (strongly agree), resulting in a total score range from 8-48. Participants were then classified into three happiness levels based on their total scores: those scoring between eight and 24 were considered to have low happiness, scores from 25 to 36 indicated moderate happiness, and scores from 37 to 48 represented high happiness. Before participating, all respondents provided written informed consent after receiving a comprehensive explanation of the study’s objectives and procedures. Participants gave written informed consent and were informed of their right to withdraw from the study at any time without providing a reason. The data were securely stored and used exclusively for research purposes.^17^,^18^

### Ethical approval:

It was obtained from the Research Ethics Committee at the Armed Forces Postgraduate Medical Institute (AFPGMI), Pakistan (Ref: 597-AAA-ERC-AFPGMI), dated April 17, 2025.

### Statistical analysis:

To analyze the data, SPSS version 27 was used. Descriptive statistics, Chi-square tests, and Ordinal Logistic Regression were used for data analysis. A p-value < 0.05 was considered statistically significant.

## RESULTS

A total of 302 nursing students participated in the study, with the majority being female (87.4%) and single (97.7%). Nearly half (49.3%) were in the second academic year. Most resided in hostels (77.8%), and 57.9% had personally chosen the nursing profession.

Associations between demographic, psychosocial, institutional, and lifestyle variables and happiness levels by using Chi-Square test is summarized in Table II-VI. Among demographic variables, only academic year showed a significant association with happiness (p = 0.003), with second-year students reporting lower happiness levels than peers in other years.

**Table-I:** Socio-demographic Profile of Nursing Students (n = 302).

Variable	Category	n (%)
Gender	Male	38 (12.6)
	Female	264 (87.4)
Marital Status	Single	295 (97.7)
	Married	6 (2)
	Others	1(0.3)
Living Arrangement	With Family	63 (20.9)
	In Hostel	235 (77.8)
	Shared Accommodation	3 (1)
	Alone	1 (0.3)
Academic Year	1st Year	36 (11.9)
	2nd Year	149 (49.3)
	3rd Year	57 (18.9)
	4th Year	60 (19.9)
Choice of the Course	Own Choice	175 (57.9)
	Parents’ Choice	111 (36.8)
	Others’ Influence	16 (5.3)

**Table-II:** Association between demographic variables and happiness levels (n = 302).

Variable	Category	Happiness Levels			p-value
		Low Happiness n (%)	Moderate Happiness n (%)	High Happiness n (%)	
Gender	Male	7 (21.9)	28(12.1)	3(7.9)	.087
	Female	25(78.1)	204(87.9)	35(92.1)	
Marital Status	Single	32 (100%)	227(97.8)	36 (94.7)	.537
	Married	0(0)	4 (1.7)	2(5.3)	
	Others	0(0)	1 (0.4)	0 (0)	
Living Arrangement	With Family	11 (34.4)	45 (19.4)	7 (18.4)	.535
	In Hostel	21 (65.6)	183 (78.9)	31 (81.6)	
	Shared Accommodation	0 (0)	3(1.3)	0 (0)	
	Alone	0 (0)	1 (0.4)	0 (0)	
Academic Year	1st Year	1(3.1)	28 (12.1)	7 (18.4)	.003
	2nd Year	25 (78.1)	103 (44.4)	21 (55.3)	
	3rd Year	4 (12.5)	45 (19.4)	8 (21.1)	
	4th Year	2 (6.3)	56 (24.1)	2 (5.3)	
Choice of the Course	Own Choice	22 (68.8)	134 (57.8)	19 (50)	.415
	Parents’ Choice	10 (31.3)	84 (36.2)	17 (44.7)	
	Others’ Influence	0 (0)	14 (6.0)	2 (5.3)	

**Table-III:** Association between social support and happiness levels (n = 302)

Variable	Category	Happiness Level			p-value
		Low Happiness n (%)	Moderate Happiness n (%)	High Happiness n (%)	
Number of Close Friends	0	5 (15.6)	25 (10.8)	6 (15.8)	.911
	1	8 (25)	75 (32.3)	14 (36.8)	
	2	9 (28.1)	65 (28)	9 (23.7)	
	3	8 (25)	60 (25.9)	8 (21.1)	
	4 and more than 4	2 (6.3)	7 (3)	1 (2.6)	
Social Support Rating	Very Poor	7 (21.9)	12 (5.2)	6 (15.8)	.008
	Poor	4 (12.5)	10 (4.3)	1 (2.6)	
	Neutral	4 (12.5)	55 (23.7)	5 (13.2)	
	Good	6 (18.8)	60 (25.9)	9 (23.7)	
	Excellent	11 (34.4)	95 (40.9)	17 (44.7)	
Support from Faculty/Mentors	No	8 (25)	26 (11.2)	6 (15.8)	.004
	Yes	10 (31.3)	142 (61.2)	27 (71.1)	
	Maybe	14 (43.8)	64 (27.6)	5 (13.2)	

**Table-IV:** Association between stress factors and happiness levels (n = 302).

Variable	Category	Happiness Leve			p-value
		Low Happiness n (%)	Moderate Happiness n (%)	High Happiness n (%)	
Sleep Quality	Very Poor	6 (18.8)	5 (2.2)	2 (5.3)	.000
	Poor	7 (21.9)	19 (8.2)	4 (10.5)	
	Neutral	8 (25)	79 (34.1)	13 (34.2)	
	Good	7 (21.9)	80 (34.5)	9 (23.7)	
	Excellent	4 (12.5)	49 (21.1)	10 (26.3)	
Stress in the Past 6 Months	Not at all	5 (15.6)	41 (17.7)	13 (34.2)	.057
	Several days	16 (50)	131 (56.5)	13 (34.2)	
	More than half the days	3 (9.4)	28 (12.1)	3 (7.9)	
	Nearly every day	8 (25)	32 (13.8)	9 (23.7)	
Satisfaction with Nursing Education	Very Dissatisfied	7(21.9)	7 (3.0)	2 (5.3)	.000
	Dissatisfied	3 (9.4)	15 (6.5)	0 (0)	
	Neutral	14 (43.8)	69 (29.7)	5 (13.2)	
	Satisfied	3 (9.4)	81 (34.9)	13 (34.2)	
	Very Satisfied	5 (15.6)	60 (25.9)	18 (47.4)	

**Table-V:** Association between institutional support and happiness levels (n = 302).

Variable	Category	Happiness Level			p-value
		Low Happiness n (%)	Moderate Happiness n (%)	High Happiness n (%)	
Sought Mental Health Support	No	18 (56.3)	155 (66.8)	15 (39.5)	.004
	Yes	14 (43.8)	77 (33.2)	23 (60.5)	
College Environment Rating	Very Poor	11 (34.4)	18 (7.8)	3 (7.9)	.000
	Poor	8 (25)	26 (11.2)	2 (5.3)	
	Neutral	9 (28.1)	91 (39.2)	6 (15.8)	
	Good	1 (3.1)	63 (27.2)	16 (42.1)	
	Excellent	3 (9.4)	34 (14.7)	11 (28.9)	
Access to Mental Health Services	No	15 (46.9)	84 (36.2)	7 (18.4)	.044
	Not Sure	4 (8.5)	39 (16.8)	4 (10.5)	
	Yes	13 (40.6)	109 (47)	27 (71.1)	
Education Funding	Self-funded	5 (15.6)	29 (12.5)	10 (26.3)	.003
	Family Supported	14 (43.8)	45 (19.4)	5 (13.2)	
	Scholarship	13 (40.6)	157 (67.7)	22 (57.9)	
	Part-time Job	0 (0)	1 (0.4)	1 (2.6)	

**Table-VI:** Association between lifestyle habits and happiness levels (n = 302).

Variable	Category	Happiness Level			p-value
		Low Happiness n (%)	Moderate Happiness n (%)	High Happiness n (%)	
Physical Exercise Frequency	Rarely/Never	14 (43.8)	59 (25.4)	12 (31.6)	.014
	Once/twice a week	8 (25)	78 (33.6)	9 (23.7)	
	3–4 times a week	9 (28.1)	46 (19.8)	4 (10.5)	
	Daily	1 (3.1)	49 (21.1)	13 (34.2)	
Participation in Extracurricular Activities	Never	16 (50)	31 (13.4)	7 (18.4)	.001
	Occasionally	13 (40.6)	150 (64.7)	15 (39.5)	
	Frequently	1 (3.1)	31 (13.4)	12 (31.6)	
	Always	2 (6.3)	20 (8.6)	4 (10.5)	

**Table-VII:** Ordinal logistic regression predicting happiness levels among nursing students (n = 302).

Variable	Category	B	SE	OR	p-value
Gender	Male	0.65	0.28	1.32	0.664
	Female (ref)	–	–	–	–
Marital Status	Single	3.44	–0.57	0.56	0.868
	Married	3.55	1.04	2.84	0.769
	Others (ref)	–	–	–	–
Living Arrangement	With Family	3.55	–0.88	0.41	0.803
	Hostel	3.53	–1.02	0.36	0.773
	Shared	3.93	–1.74	0.17	0.658
	Alone (ref)	–	–	–	–
Academic Year	1st year	0.65	0.74	2.08	0.255
	2nd year	0.57	–0.65	0.52	0.249
	3rd year	0.55	–0.05	0.95	0.935
	4th year (ref)	–	–	–	–
Course Choice	Own	–1.83	0.75	0.15	0.014 *
	Parents	–1.37	0.76	0.25	0.07
	Other influences (ref)	–	–	–	–
No. of Close Friends	0	–0.13	1.07	0.88	0.906
	1	–0.57	1	0.56	0.567
	2	–0.58	0.98	0.55	0.553
	3	–0.51	0.97	0.59	0.594
	4 or More than 4 (ref)	–	–	–	–
Participation in Extracurricular Activities	Never	–0.65	0.67	0.52	0.33
	Occasionally	0.03	0.57	1.03	0.959
	Frequently	1.22	0.65	3.37	0.062
	Always (ref)	–	–	–	–
Social support, family, friends	Very poor	0.664	0.499	1.64	0.452
	poor	0.861	-0.995	0.36	0.248
	Neutral	0.471	0.402	1.49	0.393
	Good	0.438	0.299	1.34	0.495
	Excellent (ref)	–	–	–	–
Sleep quality	poor	–0.81	0.93	0.44	0.38
	Neutral	0.09	0.68	1.08	0.9
	good	–0.02	0.47	0.98	0.974
	Excellent	–	–	–	–
Stress in the past 6 months	Not at all	0.11	0.59	1.11	0.859
	Several days	–0.86	0.55	0.42	0.115
	More than half the days	–0.90	0.69	0.4	0.19
	Nearly every day (ref)	–	–	–	–
Physical exercise frequency	Rarely/Never	0.494	-0.562	0.57	0.256
	Once/twice a week	0.481	-0.902	0.41	0.061
	3-4 times a week	0.537	-1.32	0.26	0.014
	Daily (ref)	–	–	–	–
Availability of Professional mental health support	No	0.41	0.199	1.22	0.628
	Yes (ref)	–	–	–	–
Satisfaction level with Nurse Education Program	Very Dissatisfied	0.953	-2.387	0.09	0.012
	Dissatisfied	0.827	-1.34	0.26	0.105
	Neutral	0.544	-1.086	0.33	0.046
	Satisfied	0.474	-0.144	0.86	0.761
	Very Satisfied (ref)	–	–	–	–
Availability of Support from faculty members and mentors	No	0.616	1.085	2.95	0.078
	Yes	0.421	0.658	1.93	0.118
	Maybe (ref)	–	–	–	–
University/college environment	Very poor	0.827	-1.155	0.31	0.162
	poor	0.787	-0.753	0.47	0.338
	Neutral	0.613	-0.231	0.79	0.707
	Good	0.557	0.526	1.69	0.345
	Excellent (ref)	–	–	–	–
Access to mental health support services on campus	No	0.443	-0.581	0.55	0.19
	Not Sure	0.509	-0.384	0.68	0.451
	Yes (ref)	–	–	–	–
Funding for your education	Self-funded	1.97	-3.856	0.02	0.05
	Family supported	2.003	-5.073	0.01	0.011
	Scholarship	1.98	-4.424	0.01	0.025
	Part-time job (ref)	–	–	–	–

OR: Odds Ratio; CI = Confidence Interval; SE = Standard Error.

* p < 0.05 indicates statistical significance. “ref” = reference category.

Regarding psychosocial factors, perceived quality of social support (p = 0.008) and faculty or mentor support (p = 0.004) were significant, while the number of friends was not. Among stress-related and institutional variables, sleep quality (p < 0.001), satisfaction with the nursing program (p < 0.001), and positive perceptions of the college environment (p < 0.001) were significantly associated with happiness. Students accessing mental health services or reporting supportive environments were more likely to report high happiness. Lifestyle behaviors also mattered: frequent physical exercise (p = 0.014) and participation in extracurricular activities (p = 0.001) were strongly associated with greater happiness.

### Multivariate Predictors of Happiness:

Ordinal logistic regression identified several independent predictors of happiness (Nagelkerke R^2^ = 0.393, p < 0.001). Students who chose nursing by personal interest had higher odds of greater happiness (OR = 0.15, p = 0.014). Moderate physical activity (3–4 times per week) also predicted higher happiness (OR = 0.26, p = 0.014). Compared with those ‘very satisfied’ with their nursing education, neutral participants (OR = 0.33, p = 0.046) or dissatisfied (OR = 0.09, p = 0.012) had lower happiness. Finally, students funding their studies through part-time jobs showed higher happiness compared with those relying on family, scholarships, or self-funding (all p < 0.05). Other variables were not significant predictors.

## DISCUSSION

This study examined the key determinants of happiness among nursing students in Pakistan by exploring demographic, psychosocial, institutional, and lifestyle factors. The findings provide valuable insights into the elements influencing students’ subjective well-being and highlight modifiable areas for intervention within nursing education.

Among demographic variables, the academic year emerged as a significant determinant of happiness. Second-year students were more likely to report low happiness, consistent with prior research indicating that stress peaks during mid-program stages due to academic intensity and initial clinical exposure.^19^-^22^ These findings underscore the need for early academic and emotional support systems to mitigate the psychological strain associated with clinical transition periods. In contrast, gender, marital status, and living arrangement were not significantly associated with happiness, though female students reported slightly higher well-being, a trend variably observed across cultures.^23^,^24^

Psychosocial factors played a crucial role in predicting happiness. Students who perceived strong social and faculty support reported significantly higher happiness, reinforcing the buffering effect of supportive relationships on academic stress as shown in previous studies.^25^,^26^ Interestingly, the quality of support, rather than the number of social connections, appeared to be most influential, suggesting that meaningful mentorship and empathetic peer relationships foster greater well-being.

Institutional and lifestyle variables also showed strong associations with happiness. Students reporting good sleep quality, satisfaction with their academic program, and positive perceptions of their college environment demonstrated higher levels of happiness, corroborating prior research linking academic satisfaction and institutional climate to student well-being.^24^,^26^ Access to mental health services further enhanced happiness, possibly due to early psychological intervention and stress management. Moreover, engagement in regular physical activity and extracurricular involvement were positively correlated with happiness, echoing evidence that active participation and movement improve mood and resilience.^27^ Even modest exercise showed beneficial trends, highlighting the potential of lifestyle interventions in nursing programs.

Multivariate analysis identified personal choice of profession, satisfaction with nursing education, physical activity, and mode of financial support as significant predictors of happiness. Students who selected nursing voluntarily reported higher happiness, consistent with findings linking intrinsic motivation to greater academic satisfaction, professionalism and mental well-being.^28^,^29^ Similarly, students who were satisfied with their academic experience exhibited stronger happiness levels, reflecting the influence of supportive curricula and faculty engagement.^30^ Financial independence through part-time work also predicted greater happiness, possibly due to increased autonomy and self-efficacy.^31^,^32^ Conversely, students who are dependent on family or scholarships reported lower happiness, suggesting that dependency or academic obligations may contribute to psychological strain.

Some factors, such as gender, marital status, and social support, were not significant in the regression model, diverging from trends observed in international studies.^1^,^2^,^12^ This inconsistency likely reflects sociocultural and institutional differences unique to Pakistani nursing education, including hierarchical academic structures and limited mental health infrastructure.

Collectively, these findings reveal that happiness among nursing students is shaped by an interplay of personal agency, institutional environment, lifestyle choices, and perceived control. Promoting supportive educational environments, autonomy in career selection, and healthy living habits may enhance both academic performance and emotional resilience among future nurses.

### Strengths:

This study is among the few in Pakistan to explore determinants of happiness among nursing students using **ordinal logistic regression**, which accounts for the ordered nature of happiness levels. By incorporating demographic, psychosocial, institutional, and lifestyle factors, the study provides a comprehensive perspective on student well-being and offers evidence relevant to low- and middle-income educational contexts.

### Limitations:

The cross-sectional design limits causal interpretation of associations, and self-reported measures may have introduced response or social desirability bias. The sample was restricted to a few institutions in Rawalpindi and Islamabad, which may affect generalizability to other regions or academic programs. Furthermore, unexamined variables such as personality traits, coping styles, and family dynamics may also influence happiness and should be considered in future studies.

## CONCLUSION

This study contributes to the growing literature on nursing student well-being by identifying the primary determinants of happiness in a South Asian context. Academic satisfaction, voluntary career choice, physical activity, and financial independence emerged as key predictors of happiness. These findings suggest that nursing institutions should foster environments that promote student autonomy, encourage moderate physical activity, and integrate well-being initiatives into academic programs. Policies that facilitate student-centered learning and provide meaningful part-time employment opportunities may further enhance happiness and resilience.

### Authors Contribution:

**SK, AA & AF** conceived, designed and did statistical analysis & editing of manuscript, is responsible for integrity of research.

**SK AA AS:** Literature search, data collection and manuscript writing.

All authors have read and approved the final manuscript.
